# Role of health digital twins in oncology drug development - a primer

**DOI:** 10.3389/fonc.2026.1885797

**Published:** 2026-08-24

**Authors:** Maria Garcia Requesens, Arun K. Mankan, Luca Cantini, Marco Calabresi, Laura Vidal, Joan Brown, Steven Lang, Bobby Kaura, Kirill Veselkov, Ioannis Xenarios, Kamal S. Saini

**Affiliations:** 1 Fortrea Inc., Durham, NC, United States; 2 Clinical Surgery, Keck School of Medicine, Los Angeles, LA, United States; 3 Pathway Bio, London, United Kingdom; 4 Department of Computational Medicine, Imperial College, London, United Kingdom; 5 TwinEdge Biosciences, Lausanne, Switzerland; 6 University of Lausanne, Lausanne, Switzerland; 7 Department of Medical Oncology, Cambridge University Hospitals NHS Foundation Trust, Cambridge, United Kingdom

**Keywords:** artificial intelligence, drug development, health digital twins, oncology clinical trials, precision oncology

## Abstract

Health Digital Twins (HDTs) have attracted increasing interest as a potential tool for improving both patient care and clinical research, particularly in oncology where clinical trials remain slow, costly, and operationally complex. This primer introduces the concept of digital twins in a form accessible to professionals involved in oncology clinical research and outlines the main components of health digital twins, describes how these systems are generated, and highlights their potential relevance to oncology trials. HDTs are personalized, dynamically updated, predictive, and decision−informing computational representations that integrate clinical records, laboratory measurements, imaging, pathology, and molecular data. The generation of an HDT begins with the integration of multiple sources of information to create a digital representation that reflects the patient’s disease state and can be updated as new information becomes available. These systems may be built using data−driven AI models, mechanistic physiological models, or hybrid approaches that combine both. Emerging HDT−based approaches have been proposed as a means of addressing several of the structural limitations of oncology trials by enabling in−silico experimentation, improved biological stratification of patients, and more efficient use of longitudinal real−world and historical data. In early−phase studies, HDTs can support dose selection, safety evaluation, and cohort selection, while in later phases they may contribute to optimized trial designs, synthetic control arms, and adaptive trial methodologies. These applications may reduce patient enrolment time, improve efficiency, and support simulation−driven decision−making. Despite this promise, the application of HDTs remains associated with technical, ethical, regulatory, and data−governance challenges, including data quality, model validity, bias, and uncertainty. Regulatory agencies currently view HDTs as complementary tools within model−informed drug development rather than as replacements for conventional clinical evidence. In conclusion, HDTs have the potential to transform clinical trials by enabling personalized treatments, optimizing trial design, and facilitating predictive analytics, although their applications remain emerging and require further validation, governance frameworks, and regulatory alignment to support widespread adoption.

## 1 Digital twin and health digital twin

### 1.1 Introduction to the concept of digital twin

Clinical research is necessary for the development of new treatments for cancer and other diseases but it remains slow, costly, and operationally complex. In oncology, these challenges are particularly acute because trials increasingly target molecularly defined subgroups, often require multimodal biomarker assessment, and face persistent barriers to recruitment and retention. Recent US estimates suggest that participation in cancer treatment trials is about 7.1%, although participation across all forms of cancer clinical research is higher when registries, biobanking, and other study types are included (1).

The need for more efficient oncology development is also reflected in drug-development attrition. Analyses of clinical development pipelines have shown that oncology remains one of the more difficult therapeutic areas to progress successfully from early clinical testing to approval. Hay and colleagues reported low overall clinical development success for investigational drugs, and subsequent oncology-specific analyses have continued to emphasise the limited transition of many phase I anticancer programmes into later-stage development or approval (2, 3). These observations provide a practical rationale for exploring approaches such as model-informed development, virtual cohorts, and digital twins as tools to improve prioritisation, dose selection, protocol design, and evidentiary efficiency rather than as replacements for conventional clinical evidence (4–7).

Against this background, digital twin technology has attracted growing interest as a possible way to improve both clinical care and clinical research. According to the National Academies of Sciences, Engineering, and Medicine (NASEM), a digital twin is a set of virtual information constructs that mimics the structure, context, and behaviour of a real system, is dynamically updated with data from its physical counterpart, has predictive capability, and informs decisions; bidirectional interaction between the physical and virtual entities is central to the concept (8).

In healthcare, this concept has been adapted into the idea of the Health Digital Twin (HDT): a personalised computational representation of an individual, organ, disease process, or health trajectory that is updated using relevant real-world data and used to simulate likely outcomes under different scenario (8–10). HDTs may integrate clinical records, laboratory measurements, imaging, pathology, physiological monitoring, genomics, and other -omics data (9–11). Depending on the use case, these systems may be built using data-driven AI models, mechanistic physiological models, or hybrid approaches that combine both (9–12). HDTs have attracted increasing interest as a potential tool for improving both patient care and clinical research (9, 10, 13). In oncology, examples of evidence-generating work now include image-based cancer risk prediction studies, computational radiotherapy digital twin frameworks, mechanistic tumour-growth models, virtual or synthetic control approaches, and population pharmacokinetic virtual control groups (6, 7, 12, 14, 15). However, the field is still evolving, and definitions, technical approaches, and levels of validation remain variable across the literature (9, 10, 13).

This review introduces the concept of digital twins in a form accessible to professionals involved in oncology clinical research. It outlines the main components of health digital twins, describes how these systems are generated, and highlights their potential relevance to oncology trials.

#### 1.2 What a health digital twin is - and is not

Many people use the term “digital twin” loosely to describe any medical AI. To clearly understand this technology, we need to define how data flows between the real patient and the virtual system (the computation) (8, 9, 16). A digital model is a computational representation of a system, but it may be static and may not update automatically with real-world data. In oncology, this could be a tumour growth model or an AI algorithm trained on historical imaging data to predict treatment response. A digital shadow goes a step further by allowing one-way data flow from the real system to the model; for example, a model that is periodically updated with new CT scans, laboratory results, or adverse- event data during treatment, but does not actively guide management, would be better described as a digital shadow.

A Health Digital Twin (HDT), in the stricter sense, is personalised, dynamically updated, predictive, and decision-informing, with a meaningful connection between the physical individual and the virtual representation (9). It thus relies on a continuous, two-way data flow in which information from the patient, tumour, organ, or treatment process updates the computational representation, and the representation informs a defined decision context and can for example, guide the patient’s real-world care. As an example of this, a digital twin might track a patient’s tumour using weekly blood tests. If the twin predicts the tumour will soon resist the current trial drug, it alerts the doctor to switch treatments before the tumour can grow. The patient updates the twin, and the twin changes the patient’s treatment. Because of this specific two-way loop, a digital twin is not the same as a virtual patient. Virtual patients or cohorts simulate groups of people based on averages, like a synthetic control arm in a trial, but they are not tied to one specific, living person.

Finally, a digital twin is therefore not synonymous with every AI model, every virtual patient, or every synthetic cohort. While AI powers these systems, a standalone AI tool - like a program that scans a single photo to detect melanoma - is not a twin because it lacks that continuous, back-and-forth connection to the patient (13).

Digital twins need not always be purely AI-based: the concept predates current machine-learning approaches and may rely entirely on deterministic, mechanistic, physiological, pharmacometric, or hybrid computational models when these models are linked to a defined physical counterpart and decision problem.

For the purposes of this review, the term HDT is used broadly to include patient-level, tumour-level, organ-level, population-level, and trial-process-level representations relevant to oncology drug development. This broader framing is important because oncology development may require models of disease trajectories, tumour microenvironments, radiotherapy dose-response, pharmacokinetic/pharmacodynamic exposure, treatment sequencing, or trial-operational processes, not only whole-patient twins (see **Table 1**).

**Table 1:** Spectrum of digital twin applications in oncology.

Level of twin	Physical counterpart	Illustrative oncology use	Representative evidence
Tumour-level	Individual tumour or tumour microenvironment	Tumour growth, response, resistance or immune interaction modelling	Mechanistic and semi-mechanistic tumour-growth models (12, 17)
Organ-level	Organ or anatomical treatment target	Radiotherapy planning, dose-response, toxicity optimization	High-grade glioma and prostate radiotherapy digital twin frameworks (15, 18)
Patient-level	Individual patient with longitudinal multimodal data	Longitudinal disease trajectory, treatment selection and safety prediction	Image-based risk prediction and patient-specific digital twin concepts (14, 18, 19)
Population or control-arm level	Synthetic or external control population	Synthetic controls, virtual control groups and control-arm augmentation	Population PK virtual controls and prognostic covariate adjustment approaches (7, 20, 21)
Trial-process level	Trial design, eligibility, monitoring and adaptive rules	Protocol simulation, recruitment, adaptive monitoring and operational decision support	In-silico trial and digital twin perspectives linked to emerging trial-design applications (20, 21)

#### 1.3 Generation of health digital twins

**Figure 1:**
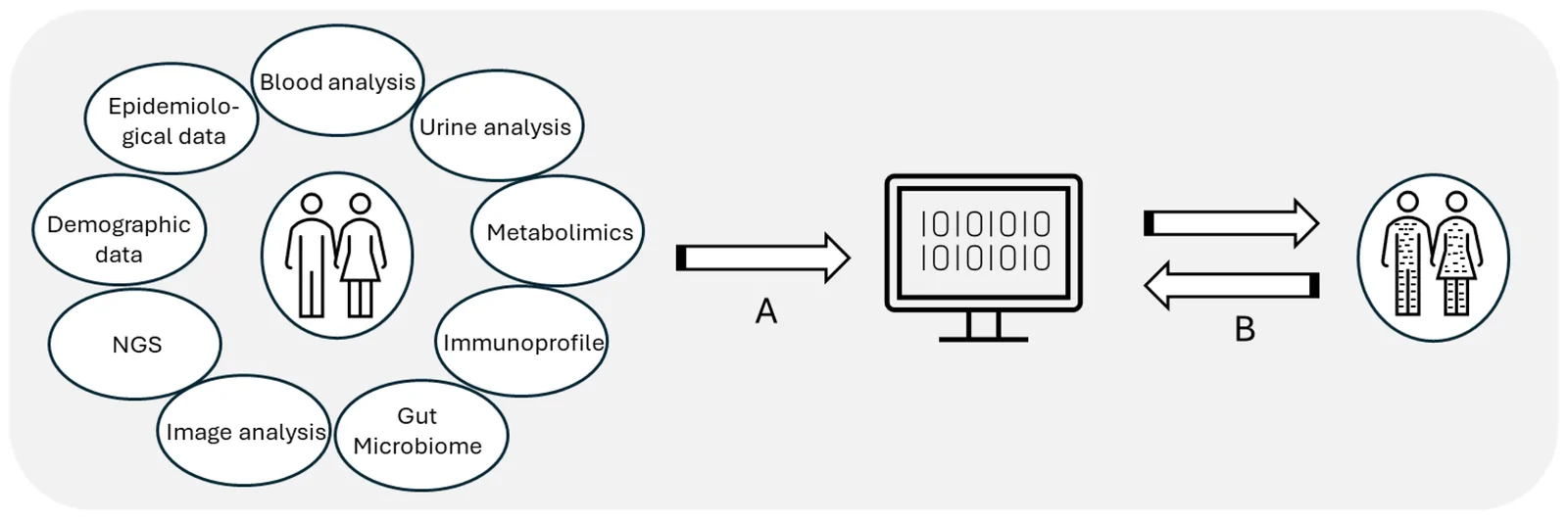
Data from multiple sources described on the left are transmitted to the computational model or digital representation **(A)**. In the earlier phases of DT model development, the outputs from the computational system needs verification and validation. As more data is captured, the reliability of the output may increase. At some point the data flow between the system and the physical individual becomes two ways i.e. The system can predict the future outcomes too **(B)**; NGS, Next Generation Sequencing.

The generation of an HDT begins with the integration of multiple sources of information. These may include demographic and clinical data, laboratory results, imaging, pathology, treatment history, physiological measurements, and molecular data such as genomics, transcriptomics, proteomics, metabolomics, or microbiome profiles (see **Figure 1**). The aim is to create a digital representation that reflects the patient’s disease state as accurately as possible and can be updated as new information becomes available. In oncology, the most informative HDTs are likely to be multimodal and longitudinal, because tumour biology, treatment response, and toxicity evolve over time and depend on interactions across several biological and clinical scales (9, 13, 22).

In practical terms, HDT generation usually starts with a foundation model built from historical or population-level data. These data help define the disease trajectories, treatment-response patterns, and biological relationships that the model needs to capture. The next step is personalisation, in which the model is adapted using data from an individual patient. For example, in oncology this might involve combining baseline CT or MRI findings, tumour histology, molecular profiling, and prior treatment history to create a patient-specific representation of the cancer. As new data are collected during treatment, such as repeat imaging, blood counts, circulating tumour DNA measurements, or patient-reported symptoms, the model can be recalibrated so that its predictions remain aligned with the patient’s current condition (23).

It is important to note that “real-time” updating is not essential in every case. Some HDTs may rely on continuous data streams from wearable sensors or remote monitoring devices, particularly when the goal is toxicity surveillance or physiological monitoring (9, 23). However, many clinically meaningful HDTs are updated periodically rather than continuously. In oncology, for instance, a twin designed to support treatment monitoring might be updated after each imaging assessment, pathology review, or treatment cycle rather than from minute-to-minute sensor data. A radiotherapy-oriented HDT may use planning CT, contouring data, dose distributions, and serial imaging to simulate tumour control and normal tissue toxicity, whereas an immuno-oncology twin may be updated using immune profiling, tumour burden, and treatment exposure over time. These examples illustrate that the defining feature of an HDT is not continuous sensing itself, but the ability to link patient data to a predictive digital representation that can evolve over time (11, 23).

Chaudhuri and colleagues developed a predictive digital twin framework for high-grade glioma radiotherapy, personalising a mechanistic model through Bayesian calibration with patient-specific magnetic resonance imaging data and then using the calibrated model to explore trade-offs between tumour control and radiotherapy toxicity (18). In a separate adaptive radiotherapy context, Chang and colleagues described a digital twin framework for online adaptive proton therapy planning in prostate stereotactic body radiotherapy, integrating daily anatomy updates and knowledge-based plan evaluation to reduce re-optimisation time while maintaining clinical-equivalent plan quality (15).

Artificial Intelligence can play an important role in this process, particularly in identifying patterns across large and heterogeneous datasets, combining information from different modalities, detecting clinically relevant changes, and improving short-term or long-term prediction. For example, machine-learning approaches may help integrate radiology, pathology, and genomic data to estimate the likelihood of treatment response, while mechanistic models may be better suited to simulating tumour growth, drug kinetics, or tumour–immune interactions (9, 16). The hybrid approaches, which combine data-driven methods with mechanistic or physiological modelling, may be especially useful because they offer a balance between predictive performance and biological interpretability (9, 13, 22). This is highly relevant in oncology, where clinicians often need not only a prediction, but also a plausible explanation for why a tumour is expected to respond, progress, or develop toxicity.

The clinical value of an HDT lies not simply in data aggregation, but in its ability to support simulation and decision-making. Once personalised and updated, an HDT may be used to estimate likely disease trajectories, compare potential treatment strategies, anticipate adverse effects, or identify a deviation from the expected clinical course. In oncology, this could mean simulating how a patient’s tumour might respond to different systemic therapies, estimating radiation dose effects on tumour and normal tissue, or identifying early signs that toxicity or resistance is emerging. At present, however, these applications should still be viewed as emerging rather than routine, and most remain in translational or proof-of-concept stages rather than in widespread clinical use (9, 10).

For clinicians, it may be helpful to think of HDT generation as an extension of familiar multidisciplinary oncology practice. Today, treatment decisions already draw on imaging, pathology, laboratory results, genomic testing, and clinical assessment. An HDT seeks to bring these elements together in a structured computational framework that can be updated over time and used not only to describe the patient’s current condition, but also to simulate what may happen next (9, 10). In this sense, the generation of an HDT is less about replacing clinical judgement and more about creating a data-informed model that can support it.

#### 1.4 Computational architectures of health digital twins

Current HDTs do not follow one universal computational architecture. Most systems can be understood as having several broad layers: data acquisition, data integration and harmonisation, modelling, twin execution or updating, and application/governance. The model layer may be data- driven, mechanistic, or hybrid, and the update cycle may be static, periodic, or near-real-time depending on the intended use case (see **Table 2**) (24, 25).

**Table 2:** Core computational architectures of health digital twins.

Architecture paradigm	Modelling principle	Example oncology trial application	Biological scale supported	Update behaviour	Key strengths	Key limitations
Data-driven HDT	AI/ML from multimodal patient data	Predicting response or toxicity to investigational therapy	Single or multiscale	Periodic or longitudinal	Strong predictive performance	Limited interpretability; bias risk
Mechanistic HDT	Mathematical representation of tumour biology or treatment effects	Simulating tumour growth or drug exposure in early phase trials	Usually multiscale	Typically longitudinal	Biologically interpretable; supports simulation	Requires biological assumptions
Hybrid HDT	Combination of mechanistic modelling and AI/ML	Integrating tumour kinetics, imaging, and biomarkers to predict outcomes	Multiscale	Longitudinal	Balance of interpretability and accuracy	Complex validation

Data-driven HDTs rely primarily on statistical learning and machine learning techniques to identify patterns within clinical data. These approaches are particularly suited to applications involving imaging, pathology, and genomic datasets where complex nonlinear relationships may not be fully understood biologically. Their main strength lies in predictive performance, although their interpretability may be limited when biological mechanisms are not explicitly represented (26).

Mechanistic HDTs are based on mathematical representations of biological processes such as tumour growth, drug pharmacokinetics/pharmacodynamics, radiation dose–response relationships or tumour-immune interactions. These models may exist without AI and may be particularly valuable when the decision requires biological interpretability, extrapolation beyond observed data, or simulation of alternative treatment schedules. Their limitations include reliance on simplified assumptions and the need for adequate patient- or system-specific calibration data (6, 12, 15).

Hybrid HDTs combine mechanistic modelling with data-driven approaches and are increasingly viewed as the most promising architecture for healthcare applications. By integrating biological understanding with machine learning, these models aim to improve predictive accuracy while maintaining clinical interpretability. This is particularly relevant in oncology, where treatment response depends on both measurable biological processes and complex interactions captured through large clinical datasets (9, 11, 12, 16).

Across these architectural types, there is increasing recognition that HDTs should be viewed not as single algorithms but as integrated modelling frameworks that combine data representation, prediction, simulation, and clinical interpretation. As the field matures, hybrid architectures that integrate multimodal data and biological knowledge are expected to become increasingly dominant due to their potential to balance predictive performance with clinical usability.

#### 1.5 Trustworthiness, validation, and governance

For HDTs to be clinically useful, technical promise alone is not enough. Their predictions must be reliable, transparent, and appropriate for the decision they are intended to support. Recent frameworks therefore place trustworthiness at the centre of HDT development, particularly in high- stakes settings such as oncology (27).

A practical way to assess trustworthiness is to consider four linked domains: technical validity, clinical validity, governance, and implementation readiness. Technical validity includes verification, validation, and uncertainty quantification (VVUQ): whether the model has been built correctly, whether it represents the real system well enough for its intended use, and how confident one can be in its predictions. Clinical validity asks whether performance is maintained in the intended patient population and care setting. Governance includes data provenance, interoperability, privacy, cybersecurity, fairness, and oversight of model updating. Implementation readiness concerns whether the HDT can be used safely within real clinical or trial workflows (27). For clinicians, the key question is straightforward: can this model be trusted to inform a real decision in a defined context of use? At present, most HDTs should still be viewed as an emerging decision-support framework rather than a mature replacement for clinical judgement or established research processes (see **Table 3**).

**Table 3:** Practical framework for clinicians.

Domain	Main question	Example in oncology
Technical validity	Was the model built and tested correctly?	Has a toxicity-prediction HDT been verified, validated, and assessed for uncertainty?
Clinical validity	Does it work in the intended patient group and clinical setting?	Does a lung cancer HDT developed in one centre perform reliably in another trial population?
Governance	Are data, privacy, fairness, and updates managed appropriately?	Are imaging, pathology, and ctDNA data traceable, secure, and representative?
Implementation readiness	Can the HDT be used safely within real workflows?	Can the model provide interpretable outputs in time for tumour board or trial decisions?

Model credibility depends on what the model is being asked to do: a model used only for internal hypothesis generation requires a lower evidentiary threshold than one used to support dosing, eligibility criteria, partial replacement of a control group, or patient-level treatment selection. Studies reporting virtual controls, adaptive radiotherapy twins, and mechanistic tumour modelling illustrate that validation should include calibration to observed data, sensitivity analyses, uncertainty quantification, and explicit acknowledgement of context of use (6, 12, 15).

## 2 Application of health digital twins in oncology clinical trials

Emerging HDT-based approaches have been proposed as a way of addressing some of the structural limitations of oncology clinical trials by enabling in-silico experimentation, improving biological stratification of patients, and making better use of clinical, molecular, and real-world data. Recent work has suggested that integrating multimodal patient data with mechanistic and machine learning models may support simulation-based decision making throughout the drug development process (13, 22, 27).

Before exploring the role of HDTs in oncology drug development, it is important to understand some of the challenges associated with conducting oncology clinical trials. Oncology remains one of the most difficult therapeutic areas in drug development, with only approximately 3–4% of oncology drug development programs progressing successfully from Phase I studies to regulatory approval. Tumour heterogeneity, complex disease biology, and rapidly evolving standards of care contribute to these low success rates, as well as to high attrition rates and long development timelines (28, 29).

Patient recruitment also remains a major challenge, particularly as trials increasingly focus on biomarker-defined populations. While precision oncology has improved patient selection, it has also resulted in smaller and more fragmented patient groups, making recruitment and retention more difficult (11, 30).

At the same time, advances in next-generation sequencing (NGS), transcriptomics, proteomics, metabolomics, and imaging have generated large amounts of biological and clinical data. Integrating and interpreting these datasets remains challenging, and important signals relating to response, toxicity, resistance mechanisms, or patient selection may be overlooked. This has led to growing interest in digital twin frameworks that can integrate data from multiple sources to generate a more comprehensive representation of an individual patient’s disease (31, 32).

Several authors have suggested that oncology may be one of the most promising areas for the application of digital twins because treatment decisions increasingly depend on complex molecular, clinical, and imaging data. By combining patient-specific information with mechanistic, statistical, or hybrid computational models, HDTs may support applications such as virtual clinical trial simulation, biomarker discovery, treatment optimisation, and adaptive trial design. Although many of these applications remain investigational, emerging studies have already demonstrated the feasibility of imaging-based risk prediction models (9, 14), mechanistic tumour and treatment-response modelling (12, 33), radiotherapy-oriented digital twins (15, 18), and virtual patient or synthetic control approaches for clinical trial design (6). Collectively, these studies illustrate how HDTs may complement existing clinical development approaches and support more efficient evidence generation (34, 35).

### 2.1 Relevance of the digital twin technology in oncology trials

Digital Twin technology has been proposed as a means of improving the precision, efficiency, and personalization of clinical trials of clinical trials by introducing a new level of precision, efficiency, and personalization (6, 9, 34).

A particularly promising application in oncology is the development of tumour-specific Digital Twins. These models integrate multimodal data including tumour genomics, transcriptomics, imaging, pathology, and longitudinal clinical outcomes to simulate tumour evolution, treatment response, and resistance mechanisms at the level of individual molecular subtypes.

In Phase I and Phase II trials, where safety, tolerability and efficacy are being tested, HDTs can support more efficient dose-finding and cohort selection by enabling extensive pre-trial simulation of alternative dosing regimens, schedules, and eligibility criteria. Rather than sequentially testing multiple configurations in patients, sponsors may explore thousands of scenarios and start a first- in-human study with a much clearer design. This use of HDTs will indirectly reduce patient enrolment time, as there is a more biological informed design, allow for the drug safety testing and ultimately maintain costs low (34–36).

HDTs have application over the whole drug development lifecycle (see **Table 4**). This includes early-phase applications such as dose selection, safety evaluation, biological activity assessment and cohort definition and all the way to population pharmacokinetic virtual control work in organ impairment studies that have demonstrated how model-derived control groups may be constructed and compared with observed control data, providing a practical analogue for model-supported trial design (6, 7). Mechanistic tumour-growth and drug-response models illustrated how simulation can be used to explore treatment schedules or biological assumptions before exposing patients to additional trial burden (12, 33, 37).

**Table 4:** Applications by trial phase.

Development phase	Evidence-backed potential role	Representative evidence	Balanced interpretation
Preclinical/nonclinical	Mechanistic models, NAMs, PK/PD simulation	FDA Modernization Act 2.0; tumour and chemotherapy modelling (12, 33, 38)	Supportive and hypothesis-generating unless model credibility is pre-specified and validated
Phase I-II	Dose selection, safety margins, cohort selection, organ-impairment modelling	Population PK virtual controls (7)	Useful for design optimization and contextualisation; not a substitute for safety observation
Phase II-III	Control-arm augmentation, protocol optimisation, adaptive design, virtual trials	In-silico trial and RCT digital twin perspectives (17, 34)	Requires strong validation, bias assessment, and regulatory alignment
Radiotherapy/multimodality	Patient- or organ-specific optimisation of dose and sequencing	Glioma and prostate radiotherapy digital twins (15, 18)	Promising for treatment planning but requires workflow integration and prospective clinical evaluation

Over time, we have seen HDTs play a role in the later phase studies, wherein sponsors could use a hybrid model with data for the control arm coming from a mix of enrolled subjects as well as HDTs. Indeed, both FDA and EMA have provided a favourable opinion for the application of selective Health Digital Twin models in clinical trials (20, 21, 36).

As mentioned, Late-phase applications may be equally important. Later development generates richer phase I-II data, making it possible to build more stable disease, exposure-response, safety, and treatment-sequencing models. HDTs may therefore support phase III protocol optimization, biomarker-enriched eligibility criteria, virtual clinical trial simulations, treatment-combination prioritization, and partial control-arm augmentation. This potential is especially relevant in multimodality oncology, where the number of plausible drug, radiation, surgery, and sequencing combinations may exceed what can be tested in conventional randomized trials (15, 17, 34, 35).

We hereby list some of the advantages of using HDTs in the overall clinical research industry.

1. Accelerated Drug Development: HDTs can model the biological systems affected by diseases and drug-specific mechanisms. For example, a pharmaceutical company developing a next-generation KRAS-G12C inhibitor could use HDTs in early development. The HDTs simulations informed by population-scale molecular and imaging datasets may identify narrow therapeutic windows and biologically rational patient subgroups shortening dose finding cycles and accelerating progression to Phase II evaluation. This simulation-driven approach accelerates the drug discovery process by predicting the drug’s efficacy and safety in a virtual environment before it’s tested in actual clinical trials. Consequently, this leads to faster development and faster approval of new drugs.
2. Optimized Clinical Trial Designs: HDTs enable the creation of virtual patient cohorts that accurately represent diverse demographics. Researchers can simulate different trial scenarios, include different varying dosing, patient demographics, and treatment durations, to then identify what could be the most effective trial designs. In a Phase II trial of a novel T-cell–engaging immunotherapy in TNBC, HDTs for example could be used to generate a large virtual cohort reflecting real-world variability in tumour biology, immune phenotype, biomarker expression, and prior treatment exposure. In silico simulations of alternative dosing strategies, treatment durations, randomisation ratios, and biomarker-based inclusion criteria indicate that a 2:1 randomisation ratio preserves statistical power while improving recruitment, that extended treatment duration benefits immune-inflamed tumours but not immune-desert tumours, and that PD-L1 and tumour mutational burden stratification enriches for responders. These insights inform a final trial design predicted to reduce required sample size by approximately 30%, lower projected costs by more than 20%, and increase the likelihood of meeting primary efficacy endpoints, with simulation data provided to regulators as supporting evidence for design optimisation (11, 17, 21). This optimization ensures that clinical trials are conducted with the highest likelihood of success, minimizing the time and resources required for trial execution (17, 34, 35).
3. Real-time Monitoring and Adaptive Trials: HDTs can be linked to real patients, providing continuous, real-time data streams. By integrating all the data available from all patient records, labs and any other tests run, and any type of real time monitoring devices into Digital Twins, researchers can monitor patients’ health status remotely. This real-time monitoring allows for adaptive trial designs, where the trial protocol can be adjusted dynamically based on the data streaming from the Digital Twins. This flexibility enhances the efficiency and effectiveness of clinical trials.
4. Enhanced Patient Engagement and Informed Consent: HDTs can create interactive, visual representations of the clinical trial process. Potential patients could be provided with more information about how HDT technology works. Availability of HDT technology would reduce the direct burden on the patient. Reduce number of visits would be required or less invasive procedures such as blood testing would be needed. This technology helps patients better understand the trial procedures, potential risks, and benefits, leading to informed consent. Additionally, HDTs can monitor patient adherence to the trial protocol, providing timely reminders and support, thus improving patient engagement and overall trial compliance.
5. Predictive Analytics and Risk Management: HDTs generate vast amounts of data, enabling the application of predictive analytics. These can be used to predict patient behaviour thereby influencing patient recruitment, engagement and retention. By analysing historical trial data and real-time information from Digital Twins, researchers can predict potential challenges, adverse events, and patient responses. This proactive approach to risk management allows for early intervention and especially well-tailored mitigation strategies, ensuring the adherence and safety of participants and the integrity of the trial.

## 3 Pitfalls and challenges of using DT

Like every novel technology, the application of HDTs has its own pitfalls. Some of the challenges in using HDTs are dependent on the underlying processes used. There are still concerns about using this technology as well as the reliability on AI systems. Despite increasing interest in Digital Twin (DT) technologies, their application in oncology drug development remains associated with several technical, ethical, regulatory, and data-governance challenges that must be addressed to enable broader adoption.

- Technical challenges primarily relate to data quality, model validity, and uncertainty. Oncology Digital Twins rely on the integration of heterogeneous data sources, including imaging, molecular profiling, clinical outcomes, and real-world evidence, all of which vary in completeness, standardisation, and longitudinal depth. As with other AI-enabled systems in oncology, there is a risk of biased or non-generalizable predictions if training data are not representative or if models are applied outside their intended context of use (9, 10, 13). Regulators increasingly expect HDT models to be fit-for-purpose, with explicit verification, validation, and uncertainty quantification proportional to the decision they are intended to inform (39–41). The radiotherapy and mechanistic modelling examples described above demonstrate that models can be tailored to specific biological or anatomical settings, but also show the need for careful parameter calibration, sensitivity analysis and uncertainty quantification before model outputs can support higher-impact decisions (12, 15).
- Study design challenges, exemplified by challenges to blinding, operational role separation and bias control. The introduction of a HDT may inadvertently influence participant and investigator expectations and trial conduct (e.g. proactive risk management), thereby increasing the risk of functional or operational unblinding. This risk is amplified when HDT implementation necessitates specialized analytics or monitoring roles with differential access to model outputs, creating informational asymmetries within an otherwise blinded workflow. Moreover, HDT-supported model-based control comparisons (i.e., predicting standard-of-care outcomes rather than observing a contemporaneous control arm) can make conventional double-blind paradigms more difficult to sustain, relevant concern given evidence that inadequate blinding can bias trial estimates, including harms.
- Ethical challenges arise from the use of patient-derived data and from DT-enabled trial designs, such as synthetic control arms. Although DT approaches may reduce patient exposure to control treatments, transparency around how individual data are used and how predictions influence trial decisions is essential to support meaningful informed consent. Ensuring fairness across patient subgroups is particularly important in oncology, given the potential for data imbalance across demographic and molecular strata.
- Regulatory challenges remain a central consideration (39, 41). Neither the FDA nor the EMA currently recognise Health Digital Twins as a standalone evidentiary substitute for conventional clinical data (39, 41). However, HDTs align conceptually with model-informed drug development, and regulators have established formal pathways to explore their use as supportive or supplementary evidence. The FDA’s Model-Informed Drug Development (MIDD) Pilot Program and corresponding EMA initiatives encourage early interaction to agree on context of use, modelling assumptions, and credibility requirements (39, 41). Within these frameworks, HDT-derived outputs are generally viewed as complementary tools to inform trial design, interpretation, and decision-making, rather than replacements for randomisation (39, 41). Regulatory bodies will require better oversight over the new technologies and their impact on clinical trial processes. Regulatory bodies themselves need to include subject matter experts who can provide scientific rationale for the use of a given technology (42).

### 3.1 Regulatory and policy developments

The FDA Modernization Act 2.0 amended the Federal Food, Drug, and Cosmetic Act by replacing language referring to “preclinical tests (including tests on animals)” with “nonclinical tests” and by defining nonclinical tests to include *in vitro*, in silico, in chemico, and non-human *in vivo* approaches. The Act further specifies that nonclinical tests may include non-animal or human biology-based methods such as cell-based assays, microphysiological systems, bioprinted models, and computer models (38). This legislative change does not mean that animal data are now irrelevant, nor that computational modelling alone will automatically be accepted for first-in-human studies. Rather, it removes the assumption that animal testing is the obligatory default and creates a clearer policy basis for validated, fit-for-purpose, human-relevant methods to support investigational new drug applications when scientifically justified.

This policy shift is important because the translational limitations of animal models are increasingly recognised. The FDA’s roadmap to reducing animal testing in preclinical safety studies notes that many drugs that appear safe and effective in animals do not ultimately receive FDA approval in humans, predominantly because of safety and/or efficacy issues, and that animal-based data have been particularly poor predictors of drug success in several disease areas, including cancer (43). The same roadmap identifies New Approach Methodologies (NAMs), including human-based *in vitro* systems, organ-on-chip and microphysiological systems, computational modelling, AI/ML, physiologically based pharmacokinetic modelling, and quantitative systems pharmacology, as approaches that can improve human relevance while reducing or replacing animal use (43). For HDTs, this creates an important regulatory opening: digital twin-derived evidence can be framed as part of a broader NAM and MIDD ecosystem, particularly when the model integrates human-derived biological data, mechanistic assumptions, and clinical or real-world evidence to address a predefined drug-development question.

ICH M15 defines MIDD as the use of computational modelling and simulation methods that may integrate nonclinical data, clinical data, prior information, and drug or disease-related knowledge to generate evidence for drug development and decision-making (39–41). Importantly, ICH M15 does not treat model outputs as self-evidently reliable; it requires explicit planning and assessment of the question of interest, context of use, model influence, consequence of a wrong decision, model risk, and model impact (39–41). This is highly relevant for HDTs. For example, an HDT used to explore alternative dose-escalation scenarios may carry a lower evidentiary burden if it only supports internal programme planning, whereas an HDT proposed to justify a reduction in animal toxicology, refine first-in-human starting dose, or replace part of a concurrent control arm would require substantially stronger validation, uncertainty characterisation, and regulatory alignment. The MIDD framework therefore provides a practical language for describing not only what an HDT predicts, but how much weight that prediction should carry in a specific development decision.

For oncology drug development, a credible HDT submission should therefore predefine the model’s context of use and decision relevance. In practical terms, sponsors should specify whether the HDT is intended to support dose selection, identify biologically rational eligibility criteria, simulate tumour growth or response, predict toxicity, optimise adaptive trial rules, augment a control arm, or support mechanistic interpretation of efficacy and safety. The model should be accompanied by a prespecified Model Analysis Plan, a Model Analysis Report, clear documentation of input data, assumptions, validation strategy, uncertainty quantification, sensitivity analyses, and limitations (39–41). In line with the FDA MIDD Paired Meeting Program, early regulatory interaction is particularly important where HDT outputs are expected to carry meaningful weight in decision-making, especially for predictive or mechanistic safety evaluation, clinical trial simulation, or dose selection (44).

Data governance and security challenges include data privacy, provenance, interoperability, cybersecurity, traceability, and auditability. HDT systems require clear governance frameworks to ensure that data sources are traceable, data transformations are transparent, and model outputs are reproducible. Compliance with regional data-protection regulations and clear accountability for model updating over time are increasingly viewed as prerequisites for regulatory confidence. This is particularly important for HDTs because models may evolve as new patient data, trial data, assays, imaging methods, or external datasets become available. Sponsors should therefore define version control, revalidation triggers, model-change management, audit trails, and procedures for detecting model drift or degraded performance over time.

Another practical issue is communication and interpretability for patients, investigators, trial staff, sponsors, and regulators. HDTs may analyse large volumes of multimodal data, but their outputs must be presented in a way that supports informed, proportionate, and auditable decisions. For clinicians and trial teams, this means that HDT outputs should not be limited to opaque risk scores; they should include interpretable summaries of the prediction, uncertainty, key drivers, limitations, and recommended actions. For patients, communication should avoid overstating certainty and should clarify that HDTs support, rather than replace, clinical judgement and protocol-defined safety oversight. For regulators, outputs should be accompanied by sufficient documentation to assess whether the model is fit for purpose in its intended context of use.

Finally, global regulatory readiness varies substantially. While the FDA, EMA, and ICH have articulated increasingly structured expectations for modelling and simulation in drug development, regulatory capacity and guidance in other regions remain uneven. This heterogeneity may complicate the global implementation of HDT-enabled strategies in oncology development programmes, particularly for multi-regional trials. International alignment on principles for model evaluation, validation, reporting, data governance, and lifecycle oversight will therefore be essential if HDTs are to move from exploratory tools to robust components of global oncology drug development.

## 4 A case study: synthetic control arm in a phase II oncology trial

This case study illustrates how HDTs can be applied to create a scientifically robust synthetic control arm in oncology drug development, reducing reliance on traditional randomised controls while preserving robust standards. The example is framed around a Phase II clinical trial evaluating a novel targeted therapy in biomarker-defined advanced non-small cell lung cancer (NSCLC), a setting in which patient recruitment is often challenging and ethical concerns arise when randomising patients to standard-of-care (SOC) alone.

For each patient enrolled in the investigational arm, a corresponding HDT is generated using a combination of historical and contemporaneous data sources. Historical real-world and clinical trial datasets include patient-level data from prior Phase II and III NSCLC studies conducted under comparable eligibility criteria, covering SOC regimens relevant to the target population. These datasets include baseline demographics, tumour histology and stage, molecular alterations (e.g. EGFR, KRAS, ALK status), prior therapies, laboratory values, radiological measurements, and longitudinal outcome data such as PFS and OS. Real-time patient-specific inputs are incorporated once the participant is enrolled, including baseline imaging, molecular profiling, clinical status, and early on-treatment biomarkers where available.

The synthetic control arm is generated using a hybrid HDT framework combining data-driven and mechanistic components, consistent with the models described earlier in this manuscript. Each HDT is personalised by conditioning the model on the enrolled patient’s baseline characteristics, thereby simulating the trajectory that that same patient would be expected to follow under SOC rather than the investigational therapy.

Several validation steps are implemented to ensure that the synthetic control arm is fit for purpose. Internal validation is performed by retrospectively applying the HDT framework to completed NSCLC trials, comparing simulated SOC outcomes against observed control-arm data to assess calibration and predictive accuracy across key endpoints PFS, OS, and response rates. Sensitivity analyses evaluate the model robustness to variations in selection, data sources, and modelling assumptions. Prospective checks are conducted during the ongoing trial by comparing early observed outcomes in enrolled patients against their predicted SOC paths, ensuring that deviations remain within pre-specified uncertainty bounds.

By substituting a portion of the traditional randomised SOC arm with the validated Digital Twin-based synthetic control, the sponsor is able to reduce the number of patients required to be randomised to SOC, accelerating enrolment and lowering overall trial costs while maintaining statistical power. Importantly, the synthetic control arm is positioned as supplementary evidence rather than a complete replacement for randomisation, in line with current regulatory guidance on model-informed drug development and hybrid control designs.

*This example highlights the capacity of Digital Twins to enhance trial efficiency, support ethical study design, and provide regulators with high-quality comparative evidence, thereby accelerating the clinical development of emerging therapies.*

In conclusion, HDTs have the potential to transform clinical trials by enabling personalised treatments, accelerating drug development, optimising trial designs, enabling real-time monitoring, enhancing patient engagement, and facilitating predictive analytics (10, 17, 34, 42, 45).

On the other hand, we should be mindful of the persistent challenges that support the use of these technologies and ensure they remain key to ongoing development efforts. Challenges like safe and appropriate data storage to avoid any information leakage, the need for strong regulatory expertise and the standardisation of data collection and analysis processes which should prevent biases. Addressing these hurdles is essential to ensuring that such innovations are put in place safely, ethically, and reliably.

As this technology continues to advance, it will play a pivotal role in reshaping the future of clinical research and improving patient outcomes.

## Author’s note

All named authors meet the International Committee of Medical Journal Editors (ICMJE) criteria for authorship for this article, take responsibility for the integrity of the work as a whole, and have given their approval for this version to be published.
